# COVID-19 severity and vaccine breakthrough infections in idiopathic inflammatory myopathies, other systemic autoimmune and inflammatory diseases, and healthy controls: a multicenter cross-sectional study from the COVID-19 Vaccination in Autoimmune Diseases (COVAD) survey

**DOI:** 10.1007/s00296-022-05229-7

**Published:** 2022-10-22

**Authors:** Leonardo Santos Hoff, Naveen Ravichandran, Samuel Katsuyuki Shinjo, Jessica Day, Parikshit Sen, Jucier Gonçalves Junior, James B. Lilleker, Mrudula Joshi, Vishwesh Agarwal, Sinan Kardes, Minchul Kim, Marcin Milchert, Ashima Makol, Tamer Gheita, Babur Salim, Tsvetelina Velikova, Abraham Edgar Gracia-Ramos, Ioannis Parodis, Albert Selva O’Callaghan, Elena Nikiphorou, Ai Lyn Tan, Tulika Chatterjee, Lorenzo Cavagna, Miguel A. Saavedra, Nelly Ziade, Johannes Knitza, Masataka Kuwana, Arvind Nune, Oliver Distler, Döndü Üsküdar Cansu, Lisa Traboco, Suryo Angorro Kusumo Wibowo, Erick Adrian Zamora Tehozol, Jorge Rojas Serrano, Ignacio García-De La Torre, Chris Wincup, John D. Pauling, Hector Chinoy, Vikas Agarwal, Rohit Aggarwal, Latika Gupta, COVAD Study Group

**Affiliations:** 1 School of Medicine, Universidade Potiguar (UnP), Natal, Brazil; 2 Department of Clinical Immunology and Rheumatology, Sanjay Gandhi Postgraduate Institute of Medical Sciences, Lucknow, India; 3 Division of Rheumatology, Faculdade de Medicina FMUSP, Universidade de Sao Paulo, Sao Paulo, SP Brazil; 4 Department of Rheumatology, Royal Melbourne Hospital, Parkville, VIC 3050 Australia; 5 Walter and Eliza Hall Institute of Medical Research, Parkville, VIC 3052 Australia; 6 Department of Medical Biology, University of Melbourne, Parkville, VIC 3052 Australia; 7 Maulana Azad Medical College, 2-Bahadurshah Zafar Marg, New Delhi, Delhi 110002 India; 8 Centre for Musculoskeletal Research, Division of Musculoskeletal and Dermatological Sciences, School of Biological Sciences, Faculty of Biology, Medicine and Health, Manchester Academic Health Science Centre, The University of Manchester, Manchester, UK; 9 Neurology, Manchester Centre for Clinical Neurosciences, Northern Care Alliance NHS Foundation Trust, Salford, UK; 10 Byramjee Jeejeebhoy Government Medical College and Sassoon General Hospitals, Pune, India; 11 Mahatma Gandhi Mission Medical College, Navi Mumbai, Maharashtra India; 12 Department of Medical Ecology and Hydroclimatology, Istanbul Faculty of Medicine, Istanbul University, Capa-Fatih, 34093 Istanbul, Turkey; 13 Center for Outcomes Research, Department of Internal Medicine, University of Illinois College of Medicine, Peoria, IL USA; 14 Department of Internal Medicine, Rheumatology, Geriatrics and Clinical Immunology, Pomeranian Medical University in Szczecin, ul Unii Lubelskiej 1, 71-252 Szczecin, Poland; 15 Division of Rheumatology, Mayo Clinic, Rochester, MN USA; 16 Rheumatology Department, Kasr Al Ainy School of Medicine, Cairo University, Cairo, Egypt; 17 Rheumatology Department, Fauji Foundation Hospital, Rawalpindi, Pakistan; 18 Department of Clinical Immunology, Medical Faculty, University Hospital “Lozenetz”, Sofia University St. Kliment Ohridski, 1 Kozyak Str., 1407 Sofia, Bulgaria; 19 Department of Internal Medicine, General Hospital, National Medical Center, La Raza”, Instituto Mexicano del Seguro Social, Av. Jacaranda S/N, Col. La Raza, Del. Azcapotzalco, 02990 Mexico City, Mexico; 20 Division of Rheumatology, Department of Medicine Solna, Karolinska Institutet and Karolinska University Hospital, Stockholm, Sweden; 21 Department of Rheumatology, Faculty of Medicine and Health, Örebro University, Örebro, Sweden; 22 Internal Medicine Department, Vall D’hebron General Hospital, Universitat Autonoma de Barcelona, 08035 Barcelona, Spain; 23 Centre for Rheumatic Diseases, King’s College London, London, UK; 24 Rheumatology Department, King’s College Hospital, London, UK; 25 NIHR Leeds Biomedical Research Centre, Leeds Teaching Hospitals Trust, Leeds, UK; 26 Leeds Institute of Rheumatic and Musculoskeletal Medicine, University of Leeds, Leeds, UK; 27 Department of Rheumatology, Fondazione I.R.C.C.S. Policlinico San Matteo, Pavia, Italy; 28 Rheumatology Unit, Dipartimento di Medicine Interna e Terapia Medica, Università degli studi di Pavia, Lombardy, Pavia, Italy; 29 Departamento de Reumatología Hospital de Especialidades Dr. Antonio Fraga Mouret, Centro Médico Nacional La Raza, IMSS, Mexico City, Mexico; 30 Rheumatology Department, Saint-Joseph University, Beirut, Lebanon; 31 Rheumatology Department, Hotel-Dieu de France Hospital, Beirut, Lebanon; 32 Medizinische Klinik 3-Rheumatologie und Immunologie, Universitätsklinikum Erlangen, Friedrich-Alexander-Universität Erlangen-Nürnberg, Ulmenweg 18, 91054 Erlangen, Germany; 33 Department of Allergy and Rheumatology, Nippon Medical School Graduate School of Medicine, 1-1-5 Sendagi, Bunkyo-ku, Tokyo, 113-8602 Japan; 34 Southport and Ormskirk Hospitals NHS Trust, Southport, PR8 6PN UK; 35 Department of Rheumatology, University Hospital Zurich, University of Zurich, Zurich, Switzerland; 36 Division of Rheumatology, Department of Internal Medicine, Eskişehir Osmangazi University, 26480 Eskişehir, Turkey; 37 Philippine Rheumatology Association, St Luke’s Medical Center-Global City, Taguig, Philippines; 38 Rheumatology Division, Department of Internal Medicine, Fakultas Kedokteran, Universitas Indonesia, Jakarta, Indonesia; 39 Rheumatology, Medical Care and Research, Centro Medico Pensiones Hospital, Instituto Mexicano del Seguro Social Delegación Yucatán, Yucatán, Mexico; 40 Rheumatologist and Clinical Investigator, Interstitial Lung Disease and Rheumatology Unit, Instituto Nacional de Enfermedades Respiratorias, Mexico City, Mexico; 41 Departamento de Inmunología y Reumatología, Hospital General de Occidente and University of Guadalajara, Guadalajara, Jalisco Mexico; 42 Department of Rheumatology, Division of Medicine, Rayne Institute, University College London, London, UK; 43 Centre for Adolescent Rheumatology Versus Arthritis at UCL, UCLH, GOSH, London, UK; 44 Bristol Medical School Translational Health Sciences, University of Bristol, Bristol, UK; 45 Department of Rheumatology, North Bristol NHS Trust, Bristol, UK; 46 National Institute for Health Research Manchester Biomedical Research Centre, Manchester University NHS Foundation Trust, The University of Manchester, Manchester, UK; 47 Department of Rheumatology, Salford Royal Hospital, Northern Care Alliance NHS Foundation Trust, Salford, UK; 48 Division of Rheumatology and Clinical Immunology, University of Pittsburgh School of Medicine, Pittsburgh, PA USA; 49 Department of Rheumatology, Royal Wolverhampton Hospitals NHS Trust, Wolverhampton, WV10 0QP UK; 50 City Hospital, Sandwell and West Birmingham Hospitals NHS Trust, Birmingham, UK

**Keywords:** Autoimmune diseases, Breakthrough infection, COVID-19, Idiopathic inflammatory myopathies, SARS-CoV-2 vaccination

## Abstract

**Objectives:**

We aimed to compare the spectrum and severity of COVID-19 and vaccine breakthrough infections (BIs) among patients with IIMs, other systemic autoimmune and inflammatory diseases (SAIDs), and healthy controls (HCs).

**Methods:**

This is a cross-sectional study with data from the COVAD study, a self-reported online global survey that collected demographics, COVID-19 history, and vaccination details from April to September 2021. Adult patients with at least one COVID-19 vaccine dose were included. BIs were defined as infections occurring > 2 weeks after any dose of vaccine. Characteristics associated with BI were analyzed with a multivariate regression analysis.

**Results:**

Among 10,900 respondents [42 (30–55) years, 74%-females, 45%-Caucasians] HCs were (47%), SAIDs (42%) and IIMs (11%). Patients with IIMs reported fewer COVID-19 cases before vaccination (6.2%-IIM vs 10.5%-SAIDs vs 14.6%-HC; OR = 0.6, 95% CI 0.4–0.8, and OR = 0.3, 95% CI 0.2–0.5, respectively). BIs were uncommon (1.4%-IIM; 1.9%-SAIDs; 3.2%-HC) and occurred in 17 IIM patients, 13 of whom were on immunosuppressants, and 3(18%) required hospitalization. All-cause hospitalization was higher in patients with IIM compared to HCs [23 (30%) vs 59 (8%), OR = 2.5, 95% CI 1.2–5.1 before vaccination, and 3 (18%) vs 9 (5%), OR = 2.6, 95% CI 1.3–5.3 in BI]. In a multivariate regression analysis, age 30–60 years was associated with a lower odds of BI (OR = 0.7, 95% CI 0.5–1.0), while the use of immunosuppressants had a higher odds of BI (OR = 1.6, 95% CI 1.1–2.7).

**Conclusions:**

Patients with IIMs reported fewer COVID-19 cases than HCs and other SAIDs, but had higher odds of all-cause hospitalization from COVID-19 than HCs. BIs were associated with the use of immunosuppressants and were uncommon in IIMs.

**Supplementary Information:**

The online version contains supplementary material available at 10.1007/s00296-022-05229-7.

## Introduction

The coronavirus disease 2019 (COVID-19) pandemic and the attendant risk of infection have been a serious cause for concern among patients with idiopathic inflammatory myopathies (IIMs) and other systemic autoimmune and inflammatory diseases (SAIDs) [1]. Patients with IIMs represent a unique vulnerable subgroup, as they typically require long-term treatment with multiple immunosuppressive and immunomodulatory (IS/IM) therapy [2, 3], which is associated with impaired host response to infections [3, 4]. Patients with IIMs may also have multiple sequelae of their disease (e.g., impaired strength, lung fibrosis) and frequent comorbidities (e.g., cardiovascular disease, obesity, and diabetes) [5]. Thus, this patient subgroup may be more susceptible to COVID-19 infection and at greater risk of severe COVID-19 complications than healthy controls and most other SAIDs [6].

Large cohort studies have indeed indicated higher COVID-19 associated mortality and poorer clinical outcomes in patients with autoimmune rheumatic diseases compared to the general population [7–9], with a hospitalization rate of 58% and fatality of 7% in these patients according to a meta-analysis [10], as opposed to a hospitalization rate in intensive care unit of 11% [11] and a fatality rate of only 1% in the general population [12]. Furthermore, despite some contradictory data regarding the incidence of COVID-19 infection among patients with autoimmune rheumatic diseases versus the general population, a recent systematic review and meta-analysis of 100 studies has demonstrated that patients with rheumatic and musculoskeletal diseases have a higher rate of SARS-CoV-2 infection and increased odds of mortality [13]. COVID-19 characteristics and outcomes are usually studied in patients with SAIDs as a large group [1, 5–10, 13], but specific data about COVID-19 in the subset of IIMs patients are scarce [14].

While there is a paucity of long-term safety and efficacy data regarding COVID-19 vaccination in patients with SAIDs, current evidence suggests that the benefits of vaccination far outweigh the potential risks of adverse effects and vaccination-induced disease flares in this vulnerable patient group [15–17]. Thus, prominent medical organizations have recommended COVID-19 vaccination in patients with autoimmune rheumatic diseases [18–21]. Of major concern is the possibility of attenuated immunogenicity and, consequently, reduced efficacy of vaccines induced by the concomitant use of IS/IM therapies in these patients, which could leave them vulnerable to breakthrough COVID-19 infections [16]. Little is currently known about the incidence and the severity of breakthrough infections (BI) in patients with IIMs and other SAIDs vaccinated against COVID-19, mainly because these patients were excluded from most trials of COVID-19 vaccines [16]. Vaccine safety and efficacy data are especially limited in patients with IIMs, and scarce data on the specific risks of COVID-19 vaccination in this patient group are currently available [22].

This study aimed to compare the frequency, profile, and severity of COVID-19 infection both prior to and post-vaccination in patients with IIMs, other SAIDs, and healthy controls (HCs).

## Materials and methods

### Study design and ethics statement

This is a cross-sectional study with secondary data from the COVID-19 Vaccination in Autoimmune Diseases (COVAD) study. COVAD study is an ongoing online questionnaire-based study that evaluates COVID-19 characteristics and vaccine safety in adult participants (older than 18 years old) diagnosed with SAIDs and healthy controls [23]. Before answering the questionnaire, participants are asked to provide their informed consent and no financial support is offered for survey completion. COVAD study was approved by the local ethics committee of Sanjay Gandhi Postgraduate Institute, Lucknow (IEC Code: 2021-143-IP-EXP-39). The current manuscript is reported according to the Checklist for Reporting Results of the Internet E-Surveys [24, 25].

### Case definition

Participants answered the question “Did you ever test positive for COVID-19?” and specified the number of events and the dates of occurrence; they also stated the dates of vaccination. BI was defined as an infection occurring more than 2 weeks after receipt of a first or second dose of a COVID-19 vaccine. Though CDC currently defines BI as an infection occurring after receipt of the second dose of a COVID-19 vaccine, we included infections occurring after the first primary dose because most people globally had only received a single vaccine dose at the time of survey dissemination, and the definition of BI was still evolving [26]. Regarding diagnostic tests, we did not confirm COVID-19 serology or vaccine immunogenicity, and this is included as a limitation of the study in the Discussion section.

### Data collection and participants

Participants answered an electronic survey consisting of 36 COVID-19 and SAIDs-related questions, which included demographics, diagnosis confirmed by a physician, current disease activity status, and treatment, COVID-19 infection history (symptoms and complications like all-cause hospitalization and requirement of oxygen), COVID-19 vaccination status and adverse effects, and outcome measures as per the Patient-Reported Outcomes Measurement Information System (PROMIS) tool [27]. The survey was developed by international rheumatology experts and was disseminated on an online platform (surveymonkey.com) after pilot testing and translation into 18 languages. Over 110 physicians from 94 countries participated in the COVAD study group and supported the dissemination of the survey on social media and online patient advocacy organizations. The questions analyzed in the current study were closed-ended. Detailed methods of the COVAD study protocol have been published elsewhere [23].

Data were retrieved from April 1st 2021 to 30th September 2021. We have chosen to analyze data from this time interval because it is considered the “first wave” of the COVAD study, comprising the period that we began to disseminate the survey and the moment we had enough answers to analyze multiple scenarios and outcomes.

### Inclusion and exclusion criteria

All respondents who received at least a single dose of the COVID-19 vaccine and fully completed the survey were included in the final analysis. Duplicate responses from a single respondent were identified using electronic protocols. Participants who were not vaccinated against COVID-19 at the time of survey completion and those who did not fully answer the survey were excluded from the analysis.

### Statistical analysis

Median and interquartile range (IQR) were reported for variables with non-normal distribution. Categorical variables were presented as frequencies and proportions. When comparing the frequency of COVID-19 and the severity of COVID-19 among IIM, other SAIDs, and HC, odds ratio (OR) adjusted for age, gender, ethnicity, and stratified by country of origin was presented. We performed a univariate regression analysis to explore if age, gender, ethnicity, vaccination status, diagnosis, corticosteroid use, and immunosuppressive or immunomodulatory therapies (IS/IM) were associated with BI. The multivariable regression analysis was stratified by country of origin and adjusted for age, gender, and covariates with a *P* < 0.1 in the univariate analysis. A *P* value < 0.05 was considered statistically significant. Scale variables were compared with Mann–Whitney *U* test and regression analysis. Statistical analysis was performed using the SPSS version 26 and Software R 3.5.3 (R Core Team 2020).

## Results

### Population characteristics

**Fig. 1:**
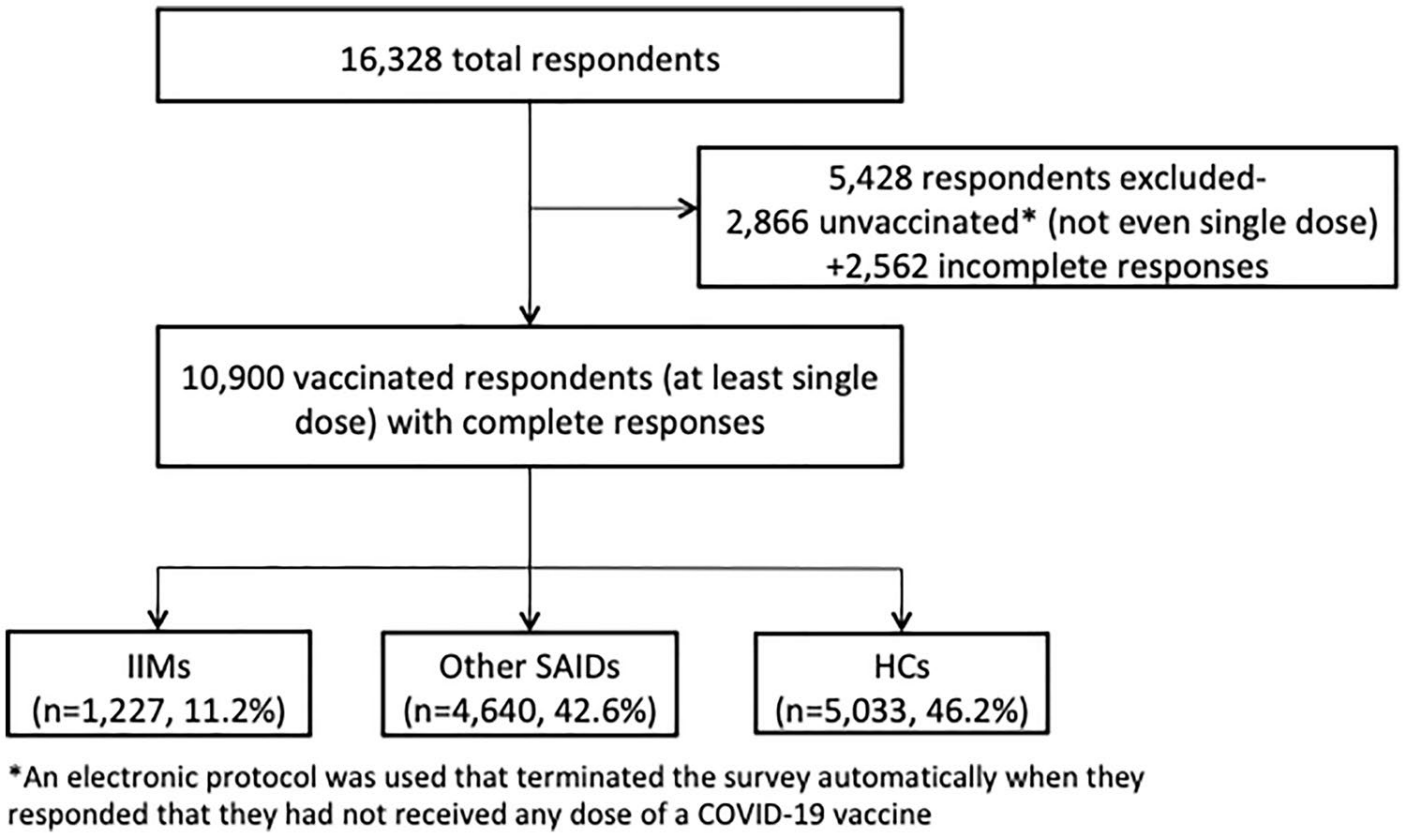
Flow diagram of study participants

Out of 16,328 respondents, 2866 did not receive any COVID-19 vaccine at the time of questionnaire fulfillment and 2562 did not entirely complete the surveyl therefore, they were excluded from the final analysis. The flow diagram with the 10,900 vaccinated respondents with complete responses included in the analysis is shown in Fig. 1. Patients with IIMs were 1227 (11.2%), while patients with other SAIDs were 4640 (42.6%), and 5033 (46.2%) were HCs. The respondents resided in 96 countries as follows: 14.9% in Turkey, 12.5% in Mexico, 13.1% in India, 11.6% in the United Kingdom, 9.9% in the United States of America, 5.5% in Italy, and 32.5% in other countries (Supplementary Table 1).

The most common SAID was rheumatoid arthritis (13%, *n* = 1459), followed by IIMs (11%, *n* = 1227), and Graves’ or Hashimoto’s disease (9%, *n* = 1051). Patients with IIMs included those with dermatomyositis, polymyositis, inclusion body myositis, antisynthetase syndrome, necrotizing autoimmune myopathy, juvenile dermatomyositis, and overlap myositis. A similar proportion of patients with IIMs (12%) and other SAIDs (13%) discontinued their IS/IM therapy prior to vaccination (Supplementary Table 2).

All respondents included in the final analysis had received at least a single dose of the vaccine at the time of survey completion and 7559 (69%) respondents had received two doses. The largest number of respondents received the BNT162b2 (Pfizer)-BioNTech vaccine (39.8%, *n* = 4333), followed by the BBIBP-CorV Sinopharm (16.7%, *n* = 1821), ChadOx1 nCOV-19 (Oxford/AstraZeneca) (13.4%, *n* = 1456), and ChAdOx1 nCoV-19 (Covishield Serum Institute India) (10.9%, *n* = 1194) vaccines (Supplementary Table 2).

The majority of COVID-19 cases occurred prior to vaccination, as seen in Table 1as follows: 1297 cases (78%) occurred prior to vaccination, 92 (6%) occurred within 2 weeks of receiving COVID-19 vaccination, and 267 (16%) occurred after the first- or second-dose of vaccination and were, therefore, considered BI. Interestingly, patients with IIMs reported fewer COVID-19 cases before vaccination (76 cases, 6.2%) than patients with other SAIDs (488 cases, 10.5%) and HCs (733 cases, 14.6%) (OR = 0.6, 95% CI 0.4–0.8, *p* = 0.002, and OR = 0.3, 95% CI 0.2–0.5, *p* < 0.001, respectively).

**Table 1:** COVID-19 cases reported before and after vaccination

COVID-19 cases: all subjects	Total (*n* = 10900)	IIMs (*n* = 1227)	Other SAIDs (*n* = 4640)	HCs (*n* = 5033)	IIMs versus Other SAIDs		IIMs versus HCs	
					OR (95% CI)	*P* value	OR (95% CI)	*P* value
Before vaccination	1297 (11.9)	76 (6.2)	488 (10.5)	733 (14.6)	0.6 (0.4–0.8)	0.002	0.3 (0.2–0.5)	< 0.001
≤ Two weeks after first or second primary vaccine dose	92 (0.8)	10 (0.8)	40 (0.9)	42 (0.8)	0.7 (0.1–2.6)	0.613	0.6 (0.2–2.2)	0.497
> Two weeks after first or second primary vaccine dose (breakthrough infection)	267 (2.5)	17 (1.4)	89 (1.9)	161 (3.2)	0.7 (0.4–1.5)	0.480	0.5 (0.2–1.1)	0.070
Total	1656 (15.2)	103 (8.4)	617 (13.3)	936 (18.6)	0.6 (0.4–0.8)	0.001	0.4 (0.3–0.5)	< 0.001

*CI* confidence interval, *HCs* healthy controls, *IIMs* idiopathic inflammatory myopathies, *OR* odds ratio, *NS* not significant, *SAIDs* systemic autoimmune and inflammatory diseases

*Odds ratio adjusted for age, gender, ethnicity, and stratified by country of origin

### Breakthrough infections (BIs)

BIs were seen in 17 (1.4%) of IIM patients, 89 (1.9%) of other SAID patients, and 161 (3.2%) of HC. Among the IIM patients with BI, nine were dermatomyositis (DM), three were anti-synthetase syndrome (ASSD), two polymyositis (PM), one overlap myositis (OM), one necrotizing autoimmune myositis (NAM), and one juvenile dermatomyositis (JDM) (Table 2). ChAdOx1 nCoV-19 (Covishield Serum Institute India) (*n* = 5) followed by BNT162b2 (Pfizer)-BioNTech vaccine (*n* = 4) takers were most frequent vaccine received by them prior to BI. Fever, fatigue, myalgia, and cough were the most common symptoms seen in them. Three (18%) were asymptomatic. The median disease duration was 7 (3–10) days. Three patients (18%) were hospitalized with or without O2 requirement following BI. Among the 89 patients with BI in SAIDs, thyroid disease (*n* = 18) and type one diabetes mellitus (*n* = 10) were the most common. Six patients (7%) were asymptomatic among them. ChAdOx1 nCoV-19 (Covishield Serum Institute India) (*n* = 24) and BNT162b2 (Pfizer)-BioNTech (*n* = 22) were the most frequent vaccine received prior to BI in them. Median disease duration was 11 (5–20) days. Ten patients (11%) had required hospitalization following BI. Among the 161 HC with BI, asymptomatic infection was seen in 20 (12%). BBIBP-CorV (Sinopharm) (*n* = 60) and ChAdOx1 nCoV-19 (Covishield Serum Institute India) (*n* = 45) were the most common vaccine received by them prior to BI. The median disease duration was 7 (3–12) days. All cause hospitalization were seen in 9 (5%) of them (Table 3).

**Table 2:** Detailed characteristics of IIMs patients with COVID-19 breakthrough infections

N	Vaccine received	Age	Gender	Country	IIMs type	Symptoms duration (days)	COVID-19 symptoms	Hospitalization or O_2_ support	IS received prior infection	Whether IS was discontinued during infection	Days between BI and vaccination
1	ChAdOx1 nCoV-19 (Covishield Serum Institute India)	45	F	India	DM	3	Fever, fatigue, myalgia, cough, diarrhea	No	None	–	19
2	BNT162b2 (Pfizer)-BioNTech	26	F	Mexico	DM	6	Headache, anosmia	No	Mtx, Aza, Prednisolone (< 10 mg/day)	Yes (Mtx for 7 days)	19
3	BNT162b2 (Pfizer)-BioNTech	62	M	USA	NAM	0	Loss of taste	No	IVIG, prednisolone (> 20 mg/day)	No	21
4	ChAdOx1 nCoV-19 (Covishield Serum Institute India)	62	F	India	DM	5	Fever, fatigue, cough, breathlessness	No	MMF, prednisolone (< 10 mg/day)	No	22
5	mRNA-1273 (Moderna)	58	F	USA	DM	8	Fever, fatigue, myalgia, cough, skin rashes	No	HCQ, Rtx	Yes (Rtx for 7 days)	25
6	ChadOx1 nCOV-19 (Oxford/AstraZeneca)	–	–	–	JDM	47	Myalgia, breathlessness	Yes	None	–	30
7	BNT162b2 (Pfizer)-BioNTech	54	F	USA	DM	7	Fatigue	No	MMF, HCQ, IVIG	No	30
8	ChAdOx1 nCoV-19 (Covishield Serum Institute India)	–	–	–	PM	11	Diarrhea, headache	Yes	Mtx	No	34
9	Coronovac	29	F	Brazil	ASSD	10	Fatigue, myalgia, chest pain, headache	No	Aza	No	35
10	ChadOx1 nCOV-19 (Oxford/AstraZeneca)	52	F	UK	ASSD	0	Fatigue, anosmia	No	Cyclo, prednisolone (> 20 mg/day)	No	47
11	mRNA-1273 (Moderna)	43	F	USA	DM	7	Fever, fatigue, cough, breathlessness, diarrhea	Yes	MMF, Rtx, prednisolone (10 mg/day)	No	48
12	BNT162b2 (Pfizer)-BioNTech	54	F	USA	OM	0	None	No	MMF, HCQ	No	48
13	ChAdOx1 nCoV-19 (Covishield Serum Institute India)	–	–	–	DM	9	None	No	Mtx	No	53
14	ChAdOx1 nCoV-19 (Covishield Serum Institute India)	–	–	–	DM	12	Fatigue	No	None	–	56
15	BBIBP-CorV (Sinopharm)	–	–	–	PM	0	None	No	None	–	57
16	BBV152 (Covaxin Bharat Biotech)	21	M	India	DM	11	Fever, fatigue, myalgia, cough	No	Mtx, prednisolone (< 10 mg/day)	No	94
17	ChadOx1 nCOV-19 (Oxford/AstraZeneca)	44	F	UK	ASSD	5	Fever, fatigue, myalgia, cough, breathlessness, chest pain, headache, nausea/vomiting	No	MMF, HCQ, prednisolone (< 10 mg/day)	Yes (MMF for 14 days)	141

*ASSD* antisynthetase syndrome, *Aza* azathioprine, *BI* breakthrough infection, *DM* dermatomyositis, *F* female, *HCQ* hydroxychloroquine, *IIM* idiopathic inflammatory myopathies, *IS* immunosuppressor, *IVIG* intravenous immunoglobulin, *JDM* juvenile dermatomyositis, *M* male, *MMF* mycophenolate mofetil, *Mtx* methotrexate, *NAM* necrotizing myositis, OM overlap myositis, *PM* polymyositis, *Rtx* rituximab, *UK* United Kingdom, *USA* United States of America

**Table 3:** COVID-19 severity in patients with idiopathic inflammatory myopathies, other autoimmune diseases, and healthy controls

Parameters	COVID-19 before vaccination			IIMs versus other SAIDs		IIMs versus HCs	
	IIMs (*n* = 76)	Other SAIDs (*n* = 488)	HCs (*n* = 733)	OR^a^ (95% CI)	*P* value	OR^a^ (95% CI)	*P* value
COVID-19 symptoms, *n* (%)	68 (89)	458 (94)	677 (92)	0.7 (0.3–1.7)	0.470	1.6 (0.5–4.7)	0.372
Different types of symptoms^b^	–	–	–	NS	NS	NS	NS
Duration of COVID-19 symptoms, median (IQR), days	10 (7, 20)	12 (7, 21)	10 (5, 15)	1.01 (0.9–1.02)	0.117	0.9 (0.9–1.1)	0.578
Asymptomatic infection	8 (11)	30 (6)	56 (8)	1.3 (0.5–3.2)	0.470	0.6 (0.2–1.7)	0.372
All-cause hospitalization and O_2_ therapy, *n* (%)	12 (16)	36 (7)	21 (3)	0.4 (0.2–1.05)	0.066	3.5 (1.3–8.9)	0.008
All-cause hospitalization (with or without O_2_ therapy), *n* (%)	23 (30)	84 (17)	59 (8)	0.7 (0.4–1.4)	0.454	2.5 (1.2–5.1)	0.011

Parameters	COVID-19 breakthrough infection^c^			IIMs versus Other SAIDs		IIMs versus HCs	
	IIMs (*n* = 17)	Other SAIDs (*n* = 89)	HCs (*n* = 161)	OR^a^ (95% CI)	*P* value	OR^a^ (95% CI)	*P* value
COVID-19 symptoms, *n* (%)	14 (82)	83 (93)	141 (88)	0.6 (0.1–8.7)	0.767	0.6 (0.2–1.4)	0.276
Headache	4 (24)	44 (49)	54 (34)	0.2 (0.1–0.9)	0.045	0.6 (0.3–1.0)	0.066
Other symptoms^b^	–	–	–	NS	NS	NS	NS
Duration of COVID-19 symptoms, median (IQR), days	7 (3, 10)	11 (5, 20)	7 (3, 12)	0.8 (0.7–0.9)	0.021	1.0 (0.9–1.1)	0.624
Asymptomatic infection	3 (18)	6 (7)	20 (12)	1.4 (0.1–1.8)	0.767	1.6 (0.6–3.7)	0.276
All-cause hospitalization and O_2_ therapy, *n* (%)	1 (6)	4 (4)	5 (3)	1.8 (0.1–2.0)	0.609	3.8 (1.5–9.0)	0.004
All-cause hospitalization (with or without O_2_ therapy), *n* (%)	3 (18)	10 (11)	9 (5)	1.5 (0.1–1.8)	0.714	2.6 (1.3–5.3)	0.006

*CI* confidence interval, *HCs* healthy control, *IIMs* idiopathic inflammatory myopathies, *IQR* interquartile range, *OR* odds ratio, *NS* not significant, *SAIDs* systemic autoimmune and inflammatory diseases

^a^Odds ratio adjusted for age, gender, ethnicity, and stratified by country of origin

^b^COVID-19 symptoms individually assessed were as follows: fever, fatigue, myalgia, cough, breathlessness, chest pain, diarrhea, headache, oral ulcers, nausea/vomiting, arthralgia, skin rashes, or others

^c^Breakthrough infection was defined as an infection occurring more than two weeks after receipt of a first or second dose of a COVID-19 vaccine

### COVID-19 severity among IIM, other SAIDs, and HCs

Patients with IIMs had higher odds of all-cause hospitalization in comparison to HCs either in COVID-19 cases that occurred prior to vaccination (OR = 2.5, 95% CI 1.2, 5.1, *p* = 0.011) or in BI (OR = 2.6, 95% CI 1.3–5.3, *p* = 0.006). Hospitalization with supplemental oxygen requirement was also higher in patients with IIMs versus HCs in both situations (prior to vaccination and BI, OR = 3.5, 95% CI 1.3–8.9, *p* = 0.008, and OR = 3.8, 95% CI 1.5–9.0, *p* = 0.004, respectively). However, all-cause hospitalization was similar in IIM in comparison to SAIDs. The specific COVID-19 symptoms and disease duration were comparable among the three groups, except for headache and the duration of breakthrough COVID-19 infections, which were less frequent and of shorter duration in IIMs than in patients with other SAIDs (OR = 0.2, 95% CI 0.1–0.9, *p* = 0.045, and OR = 0.8, 95% CI 0.7–0.9, *p* = 0.021, respectively) (Table 3).

### Characteristics associated with BIs

We assessed whether age, gender, ethnicity, vaccination status, diagnosis, corticosteroid use, and other IS/IM therapies were associated with BI. In a multivariate model adjusted for covariates with a *p* < 0.1 in the univariate analysis, the following covariates had an association with BI: age 30–60 years (OR = 0.7, 95% CI 0.5–1.0, *p* = 0.041), two or more COVID-19 vaccine doses (OR = 2.0, 95% CI 1.4–2.8, *p* < 0.001), and exposure to IS/IM therapies (OR = 1.6, 95% CI 1.1–2.7, *p* = 0.029) (Table 4).

**Table 4:** Univariate and multivariable regression analysis of characteristics associated with COVID-19 breakthrough infection

Variable	Category	Univariate		Multivariate^a^	
		OR (95% CI)	*P* value	OR (95% CI)	*P* value
Age	< 30 years	Reference	Reference	Reference	Reference
	30–60 years	1.1 (0.9–1.2)	0.098	0.7 (0.5–1.0)	0.041
	> 60 years	1.4 (0.8–2.4)	0.155	1.3 (0.7–2.2)	0.312
Gender	Male	Reference	Reference	Reference	Reference
	Female	0.8 (0.6–1.1)	0.225	0.8 (0.6–1.1)	0.379
Ethnicity	Caucasian	Reference	Reference	Reference	Reference
	Not caucasian	1.1 (0.8–1.4)	0.546	1.0 (0.7–1.4)	0.735
COVID-19 vaccination	None	–	–	–	–
	One dose	Reference	Reference	Reference	Reference
	≥ Two doses	1.5 (1.2–1.9)	< 0.001	2.0 (1.4–2.8)	< 0.001
Diagnosis	IIM	Reference	Reference	Reference	Reference
	Other SAIDs	0.8 (0.4–1.5)	0.581	0.9 (0.4–1.9)	0.957
	HC	1.0 (0.6–1.8)	0.898	0.9 (0.6–1.3)	0.882
Corticosteroid use	None	Reference	Reference	Reference	Reference
	≤ 10 mg of prednisone equivalent	0.6 (0.3–1.2)	0.149	0.6 (0.3–1.2)	0.202
	> 10 mg of prednisone equivalent	0.6 (0.2–1.7)	0.422	0.7 (0.2–2.2)	0.627
Other IS/IM therapies	None	Reference	Reference	Reference	Reference
	Yes	1.5 (1.1–2.3)	0.023	1.6 (1.1–2.7)	0.029

Breakthrough infection was defined as an infection occurring more than 2 weeks after receipt of a first or second dose of a COVID-19 vaccine

*CI* confidence interval, *HCs* healthy control, *IIMs* idiopathic inflammatory myopathies, *IS/IM* immunosuppressive and immunomodulatory therapy, *OD* odds ratio, *NS* not significant, *SAIDs* systemic autoimmune and inflammatory diseases

^a^Multivariate regression analysis was stratified by country of origin and adjusted for age, gender, and covariates with a *p* < 0.1 in the univariate analysis

## Discussion

In the present study, we found that patients with IIMs reported fewer COVID-19 infections prior to vaccination than patients with other SAIDs and HCs, yet they had higher odds for all-cause hospitalization than HCs. Vaccine BI was uncommon and their characteristics were comparable among the groups of IIMs, SAIDs, and HCs, except for headache and duration of COVID-19 symptoms, which was shorter in IIMs than in SAIDs. The factors associated with higher odds of BI infections were exposure to IS/IM therapies and more than two doses of COVID-19 vaccine, while age 30–60 was associated with lower odds of BI. Gender, ethnicity, steroid use, and diagnosis (IIM, SAID, HC) conferred similar odds of BI in the multivariate analysis.

Patients with SAIDs, including IIMs, are a vulnerable population at an increased risk of disease severity and poorer clinical outcomes related to COVID-19 infection and possibly a higher incidence of COVID-19 infection compared to healthy counterparts [10, 13, 14]. We found a reported frequency of COVID-19 of 6.2% in IIMs patients and 10.5% in SAIDs before vaccination, which is higher than the rate of 0.36% previously reported in a systematic review of patients with inflammatory and autoimmune rheumatic diseases [13]. This may be explained by the design of our study, which is prone to recall and selection bias, with patients willing to answer the e-survey more likely to have had COVID-19 infection and thus more likely to remember the symptoms. Surprisingly, the self-reported pre-vaccination incidence of COVID-19 infection was lower in patients with IIMs than in patients with other SAIDs and HCs, and the diagnosis of IIMs was associated with a smaller odds of COVID-19 than other SAIDs and HCs, possibly due to protective behaviors taken by this extremely vulnerable population like physical distancing and shielding [1].

In our study, all-cause hospitalizations in IIMs patients were higher than in patients with other SAIDs (30 versus 17%, respectively). Considering that IS/IM therapies are a known risk factor for worst outcomes in COVID-19 [9, 11, 14], our sample of patients with IIMs was more on IS/IM therapy than patients with other SAIDs, which may explain this finding. It is unclear why IIMs patients with BI had a lower incidence of headache and shorter duration of COVID-19 symptoms than patients with other SAIDs; this may represent a unique feature of COVID-19 in this subset of patients, which needs to be confirmed in future studies, or more probably a spurious finding owing to few BI cases in the IIMs group.

Exposure to IS/IM therapies was associated with higher odds of BI. Evidence suggests that IS/IM medications, particularly B-cell depleting agents, may impair the host response to COVID-19 vaccines, thus reducing their immunogenicity [15, 16]. In our study, few patients with BI discontinued their IS/IM treatment before vaccination, which may explain this finding. However, suspension of IS/IM therapy prior to COVID-19 vaccination may not always be clinically appropriate, and this is a decision that must be made on an individual basis [17–21].

Two or more COVID-19 vaccine doses were also associated with a higher odds of BI than one vaccine dose. The most reasonable explanation for this finding is this may represent a follow-up bias, as patients with two or more vaccine doses have a longer follow-up and, therefore, are more prone to BI than patients with a shorter follow-up. A recent systematic review has shown that COVID-19 vaccines are effective against infection, but this protection wanes over time, especially in mild to moderate cases [28]. Considering that most of COVID-19 cases described in our study were mild or even asymptomatic, this also may explain why so many patients with two or more vaccine doses presented with BI.

The type of vaccine received may also influence the risk of breakthrough COVID-19 infection, owing to their variable efficacy, mechanism of action, immunogenicity, adjuvants, and host interaction [16]. A large cohort of patients with rheumatic disease followed in almost the same period in 2021 has shown a BI rate of 0.9%, and half of these patients had received two or more vaccine doses; the authors did not explore possible risk factors for BIs in this study, nor could associate these cases with a specific type of vaccine [29]. The efficacy of different vaccines in preventing BI in patients with IIMs and other SAIDs needs to be evaluated in future studies with an appropriate design and long-term follow-up. The most recent recommendation from the American College of Rheumatology emphasizes the response to COVID-19 vaccination in patients with SAIDs receiving IS/IM drugs is likely to be blunted in comparison to the general population; therefore, it may explain a higher number of BIs in fully vaccinated patients with SAIDs [19].

COVAD is one of the largest studies about COVID-19 vaccination in patients with SAIDs, with a representative sample from 94 countries. This is a strength of the current study, contributing to the external validity of our findings. However, this study has limitations. Using the proper statistical analysis for a cross-sectional study (a multivariate regression analysis), we found that BIs were associated with exposure to IS/IM drugs. We acknowledge that we cannot assume a cause-and-effect relationship between IS/IM drugs and BI; therefore, this association should be further confirmed in cohort or randomized controlled trial studies. We did not disseminate our survey in a systematic way, so our population represents a convenience sample. We targeted our survey to patients with SAIDs in general, and there were no steps taken to make any subgroup of SAIDs representative. Considering the inherent profile of patients who can respond to an online survey, we may infer that low-income patients without internet access, severely disabled, and deceased are not represented in our study. Additionally, our conclusions are based on self-reported data that could not be checked on medical records. Furthermore, we did not explore several confounding variables that could impact the outcomes assessed, including protective behaviors against COVID-19 infection, education level, income, access to health services, multiple vaccine combinations, and comorbidities. Considering the small number of BI in our samples, we could not assess whether different types of vaccines influenced the risk of BI. Finally, we neither confirmed COVID-19 serology nor vaccine immunogenicity. Though we have used a broader definition for BI, this study provides unique insights into the protection offered by a single dose of vaccine against COVID-19.

In conclusion, unvaccinated patients with IIMs reported fewer COVID-19 cases in comparison to patients with other SAIDs and HCs, but were more vulnerable to all-cause hospitalization in comparison to HCs. Overall, COVID-19 BI was uncommon and was comparable among IIMs, other SAIDs, and HCs. Despite the aforementioned limitations, this study adds to the valuable understanding of COVID-19 severity and characteristics in vaccinated and unvaccinated patients with IIMs and other SAIDs.

## Supplementary Information

Below is the link to the electronic supplementary material.

Supplementary file1 (DOCX 18 KB)

Supplementary file2 (DOCX 20 KB)

Supplementary file3 (DOCX 23 KB)
