# Supplementary material for: COVID-19 severity and vaccine breakthrough infections in idiopathic inflammatory myopathies, other systemic autoimmune and inflammatory diseases, and healthy controls: a multicenter cross-sectional study from the COVID-19 Vaccination in Autoimmune Diseases (COVAD) survey
Source: Rheumatol Int. 2022 Oct 22;43(1):47–58. doi: 10.1007/s00296-022-05229-7 (PMC9589602; doi:10.1007/s00296-022-05229-7)
Supplement: Supplementary file 1 — Supplementary file1 (DOCX 18 KB) [file 296_2022_5229_MOESM1_ESM.docx]

**Supplementary Table 1.** Participants’ country of residence

| Country of Residence | Responses | | | Country of  Residence | Responses | | Country of Residence | Responses | | Country of Residence | Responses | |
| --- | --- | --- | --- | --- | --- | --- | --- | --- | --- | --- | --- | --- |
| Turkey | 14.95% | 1518 | United Arab Emirates | | 0.32% | 33 | Qatar | 0.05% | 5 | South Africa | 0.02% | 2 |
| India | 13.15% | 1335 | Australia | | 0.30% | 30 | Netherlands | 0.04% | 4 | Hungary | 0.01% | 1 |
| Mexico | 12.53% | 1272 | Bangladesh | | 0.25% | 25 | Afghanistan | 0.03% | 3 | Iraq | 0.01% | 1 |
| United Kingdom | 11.61% | 1179 | Cyprus | | 0.18% | 18 | Belgium | 0.03% | 3 | Jamaica | 0.01% | 1 |
| United States | 9.93% | 1008 | Ireland (Republic of) | | 0.17% | 17 | Czech Republic | 0.03% | 3 | Jordan | 0.01% | 1 |
| Italy | 5.50% | 559 | Argentina | | 0.16% | 16 | Greece | 0.03% | 3 | Kenya | 0.01% | 1 |
| Switzerland | 3.04% | 309 | Austria | | 0.16% | 16 | New Zealand | 0.03% | 3 | Korea South | 0.01% | 1 |
| Philippines | 2.89% | 293 | Colombia | | 0.13% | 13 | Ukraine | 0.03% | 3 | Kuwait | 0.01% | 1 |
| Brazil | 2.60% | 264 | Venezuela | | 0.12% | 12 | Albania | 0.02% | 2 | Libya | 0.01% | 1 |
| Poland | 2.60% | 264 | Kazakhstan | | 0.11% | 11 | Algeria | 0.02% | 2 | Liechtenstein | 0.01% | 1 |
| Sweden | 2.57% | 261 | Romania | | 0.11% | 11 | Antigua & Deps | 0.02% | 2 | Luxembourg | 0.01% | 1 |
| Lebanon | 2.26% | 229 | Ecuador | | 0.09% | 9 | Azerbaijan | 0.02% | 2 | Malta | 0.01% | 1 |
| Indonesia | 1.93% | 196 | Morocco | | 0.09% | 9 | Dominican Rep. | 0.02% | 2 | Micronesia | 0.01% | 1 |
| Egypt | 1.54% | 156 | Portugal | | 0.09% | 9 | Iran | 0.02% | 2 | Montenegro | 0.01% | 1 |
| Germany | 1.51% | 153 | Saudi Arabia | | 0.09% | 9 | Norway | 0.02% | 2 | Nigeria | 0.01% | 1 |
| Japan | 1.42% | 144 | Singapore | | 0.08% | 8 | Andorra | 0.01% | 1 | Oman | 0.01% | 1 |
| Pakistan | 1.33% | 135 | Chile | | 0.07% | 7 | Barbados | 0.01% | 1 | Panama | 0.01% | 1 |
| Bulgaria | 1.27% | 129 | Guatemala | | 0.07% | 7 | Cape Verde | 0.01% | 1 | Paraguay | 0.01% | 1 |
| Spain | 1.04% | 106 | El Salvador | | 0.06% | 6 | China | 0.01% | 1 | San Marino | 0.01% | 1 |
| Russian Federation | 0.74% | 75 | Finland | | 0.06% | 6 | Comoros | 0.01% | 1 | Serbia | 0.01% | 1 |
| Bahrain | 0.58% | 59 | Honduras | | 0.06% | 6 | Denmark | 0.01% | 1 | Swaziland | 0.01% | 1 |
| Israel | 0.53% | 54 | Bolivia | | 0.05% | 5 | Estonia | 0.01% | 1 | Taiwan | 0.01% | 1 |
| Canada | 0.46% | 47 | Costa Rica | | 0.05% | 5 | Gabon | 0.01% | 1 | Thailand | 0.01% | 1 |
| France | 0.41% | 42 | Malaysia | | 0.05% | 5 | Grenada | 0.01% | 1 | Trinidad & Tobago | 0.01% | 1 |
