# Supplementary material for: COVID-19 severity and vaccine breakthrough infections in idiopathic inflammatory myopathies, other systemic autoimmune and inflammatory diseases, and healthy controls: a multicenter cross-sectional study from the COVID-19 Vaccination in Autoimmune Diseases (COVAD) survey
Source: Rheumatol Int. 2022 Oct 22;43(1):47–58. doi: 10.1007/s00296-022-05229-7 (PMC9589602; doi:10.1007/s00296-022-05229-7)
Supplement: Supplementary file 2 — Supplementary file2 (DOCX 20 KB) [file 296_2022_5229_MOESM2_ESM.docx]

**Supplementary Table 2.** Population characteristics

| **Variable** | **Total**  **(n=10,900)** | **IIMs (n=1,227)** | **Other SAIDs (n=4,640)** | **HCs**  **(n=5,033)** |
| --- | --- | --- | --- | --- |
| Age, median (IQR), years | 42 (30-55) | 49 (38-61) | 47 (36-57) | 33 (25-46) |
| Gender male: female*  Gender ratio | 2432: 8558 1: 2.9 | 283: 782  1: 2.8 | 568: 3249 1: 5.7 | 1491: 2798 1: 1.8 |
| **Ethnicity, n (%)** |  |  |  |  |
| Caucasian | 4,972 (45) | 882 (71) | 2,303 (49) | 1,787 (35) |
| African American or of African origin | 83 (0.7) | 34 (3) | 22 (0.5) | 27 (0.5) |
| Asian | 2,018 (18) | 71 (6) | 781 (17) | 1,166 (23) |
| Hispanic | 1,193 (11) | 49 (4) | 399 (8) | 745 (15) |
| Native American/Indigenous/Pacific Islander | 342 (3) | 1 (0) | 18 (0.3) | 323 (6) |
| Did not wish to disclose | 449 (4) | 13 (1) | 191 (4) | 245 (5) |
| Other | 865 (8) | 21 (1.7) | 127 (3) | 717 (14) |
| **Vaccine received, n (%)**  Pfizer-BioNTech (BNT162b2)  ChadOx1 nCOV-19 (Oxford/AstraZeneca)  JNJ-78436735 (Johnson and Johnson)  mRNA-1273 (Moderna)  Novavax (NVX-CoV2373)  ChAdOx1 nCoV-19 (Covishield SII)  BBV152 (Covaxin Bharat Biotech)  Gam-COVID-Vac (Sputnik)  BBIBP-CorV (Sinopharm)  Did not know  Others | 4,333 (39)  1,456 (13) 95 (1)  910 (8)  14 (0.1)  1,194 (11)  248 (2)  204 (2)  1,821 (17)  62 (0.5)  563 (5) | 645 (53)  124 (10)  15 (1.2)  360 (29)  0 (0)  43 (3.5)  15 (1.2)  4 (0.3)  4 (0.3)  0 (0)  17 (1.4) | 2,042 (44)  845 (18)  42 (1)  387 (8)  10 (0.2)  430 (9)  111 (2)  64 (1)  374 (8)  27 (0.5)  309 (6) | 1,443 (28.7)  487 (9.7)  38 (0.8)  163 (3.2)  4 (0.1)  721 (14)  122 (2.4)  136 (2.7)  1,443 (28.7)  35 (0.7)  238 (4.7) |
| **Diagnosis, n (%)**  No autoimmune disease  Dermatomyositis  Polymyositis  Inclusion body myositis  Anti-synthetase syndrome  Necrotizing myositis  Juvenile dermatomyositis  Mixed connective tissue disorder  Overlap myositis with other CTD  Systemic sclerosis  Sjogren’s syndrome  Rheumatoid arthritis  Vasculitis  Systemic lupus erythematosus  Ankylosing spondylitis or PsA  Crohn’s disease or ulcerative colitis  Multiple sclerosis  Myasthenia gravis  Pernicious anemia  Hemolytic anemia/ITP  Hashimoto’s or Graves’ disease  Type one diabetes mellitus  Polymyalgia rheumatica  Others | 5,033 (46)  418 (4)  207 (2)  284 (2)  136 (1)  52 (0.5)  14 (0.1)  106 (1)  116 (1)  493 (4)  294 (3)  1,459 (13)  142 (1)  600 (6)  394 (4)  239 (2)  46 (0.5)  46 (0.5)  24 (0.2)  32 (0.2)  1,051 (9)  141 (1)  43 (0.3)  1,192 (10) | -  418 (39)  207 (23)  284 (23)  136 (11)  52 (5)  14 (1.4)  -  116 (11)  -  -  -  -  -  -  -  2 (0.2)  6 (0.5)  3 (0.2)  4 (0.3)  168 (13.5)  17 (1.4)  3 (0.2)  131 (10) | -  -  -  -  -  -  -  106 (2)  -  493 (11)  294 (6)  1,459 (31)  142 (3)  600 (13)  394 (8)  239 (5)  44 (0.9)  40 (0.9)  21 (0.5)  28 (0.6)  883 (19)  124 (3)  40 (1)  1,061 (22) | 5,033 (100)  -  -  -  -  -  -  -  -  -  -  -  -  -  -  -  -  -  -  -  -  -  -  - |
| **IS/IM therapy, n (%)**  Methotrexate  Mycophenolate mofetil  Azathioprine  Rituximab  Hydroxychloroquine  Sulfasalazine  Leflunomide  Calcineurin Inhibitors  Intravenous Immunoglobulin  Cyclophosphamide  No corticosteroids  <10 mg/day prednisone equivalent  10-20 mg/day steroids prednisone equivalent  >20 mg/day steroids prednisone equivalent | 1,155 (10)  502 (4)  324 (3)  102 (1)  1,002 (9)  222 (2)  174 (1.5)  107 (1)  157 (1.4)  19 (0.1)  3,901 (36)  1,134 (10)  241 (2)  99 (1) | 252 (20)  224 (18)  112 (9)  40 (3)  179 (14)  14 (1)  20 (1.6)  54 (4)  117 (9)  6 (0.4)  676 (55)  359 (29)  84 (7)  39 (3) | 903 (19)  278 (6)  212 (4)  62 (1)  823 (17)  208 (4)  154 (3)  53 (1)  40 (0.8)  13 (0.2)  3,225 (69)  775 (16)  157 (3)  60 (1) | -  -  -  -  -  -  -  -  -  -  -  -  -  - |
| Discontinued IS/IM before vaccination, n (%) | 773 (7) | 147 (12) | 626 (13) | - |
| Duration of discontinuing IS/IM, median (IQR), days | 13 (7, 21) | 14 (7, 21) | 12 (7, 21) | - |

Abbreviations: CTD, connective tissue diseases; HCs, healthy controls; IIMs, idiopathic inflammatory myopathies; IS/IM, immunosuppressive and immunomodulatory therapy; ITP, idiopathic thrombocytopenic purpura; PsA, psoriatic arthritis; SAIDs, systemic autoimmune and inflammatory diseases; SII, Serum Institute of India.

*The remaining respondents did not wish to disclose their gender.
