# Supplementary material for: COVID-19 severity and vaccine breakthrough infections in idiopathic inflammatory myopathies, other systemic autoimmune and inflammatory diseases, and healthy controls: a multicenter cross-sectional study from the COVID-19 Vaccination in Autoimmune Diseases (COVAD) survey
Source: Rheumatol Int. 2022 Oct 22;43(1):47–58. doi: 10.1007/s00296-022-05229-7 (PMC9589602; doi:10.1007/s00296-022-05229-7)
Supplement: Supplementary file 3 — Supplementary file3 (DOCX 23 KB) [file 296_2022_5229_MOESM3_ESM.docx]

**Supplementary File 3**. COVID-19 Vaccination in Autoimmune Diseases (COVAD) Study Group Author List and Affiliations

**India**

1. **Dr Bhupen Barman**, Department of Medicine, North Eastern Indira Gandhi Regional Institute of Health and Medical Sciences (NEIGRIHMS), Shillong-18, Meghalaya, India
2. **Dr Yogesh Preet Singh**, Division of Rheumatology and Clinical Immunology, Department of General Medicine, Himalayan Institute of Medical sciences, Swami Rama University, Jolly Grant, Dehradun - 248140, Uttarakhand, India
3. **Dr Rajiv Ranjan**, Clinical Immunology & Rheumatology at Columbia Asia, Palam Vihar, Gurgaon, Haryana, India
4. **Dr Avinash Jain**, Department of Clinical Immunology and Rheumatology, SMS Medical College and Hospital, Jaipur, Rajasthan, India
5. **Dr Sapan C Pandya**, Clinical Immunology and Rheumatology, Rheumatic Disease Clinic, Vedanta Institute of Medical Sciences, Navrangpura, Ahmedabad 380009, Gujarat, India
6. **Dr Rakesh Kumar Pilania**, Pediatric Allergy Immunology Unit, Department of Pediatrics, Post Graduate Institute of Medical Education and Research, Chandigarh, India
7. **Dr Aman Sharma**, Clinical Immunology and Rheumatology Services, Department of Internal Medicine, Post Graduate Institute of Medical Education and Research, Chandigarh, India
8. **Dr Manesh Manoj M**, Department of Clinical Immunology and Rheumatology, AKG Memorial Hospital and Dr Shenoy’s CARE (Centre for Arthritis and Rheumatism Excellence), Kannur, Kerala, India
9. **Dr Vikas Gupta**, Rheumatology, Dayanand Medical College and Hospital, Ludhiana, Punjab 141001, India
10. **Dr Chengappa G Kavadichanda**, Department of Clinical Immunology, Jawaharlal Institute of Postgraduate Medical Education and Research, Puducherry, India
11. **Dr Pradeepta Sekhar Patro**, Department of Clinical Immunology and Rheumatology, Sunshine Hospitals, Plot No 208, Cuttack Puri Road, Laxmisagar, Bhubaneshwar, Odisha, India
12. **Dr Sajal Ajmani**, Arthritis and Rheumatology clinic, New Delhi, Delhi, India
13. **Dr Sanat Phatak**, Department of Rheumatology and Immunology, KEM Hospital, Pune, Maharashtra, India
14. **Dr Rudra Prosad Goswami**, Department of Rheumatology, All India Institute of Medical Sciences, New Delhi, Delhi, India
15. **Dr Abhra Chandra Chowdhury**, AMRI Hospital, Dhakuria, Kolkata, West Bengal, India
16. **Dr Ashish Jacob Mathew**, Department of Clinical Immunology & Rheumatology, Christian Medical College and Hospital, Vellore, Tamil Nadu 632004, India
17. **Dr Padnamabha Shenoy**, Dr Shenoy’s CARE (Centre for Arthritis and Rheumatism Excellence), Kannur, Kerala, India
18. **Dr Ajay Asranna**, Department of Neurology, NIMHANS, Bengaluru, Karnataka, India
19. **Dr Keerthi Talari Bommakanti**, Yashoda hospital, Behind Hari Hara Kala Bhavan, Secunderabad - 500003, T.S. Hyderabad, Telangana, India
20. **Dr Anuj Shukla**, Niruj Rheumatology Clinic, 209 Rajvi Complex, Rambaug, Ahmedabad, 380008, Gujarat, India
21. **Dr Arun Kumar R Pandey,** LEDTC Clinic, Gomti Nagar, Lucknow, Uttar Pradesh, India
22. **Dr Kunal Chandwar**, Department of Clinical Immunology and Rheumatology, King George's Medical University, Lucknow, Uttar Pradesh, India.

**Italy**

1. **Dr. Nicoletta Del Papa**, Unità operativa complessa (UOC) Day Hospital Reumatologia via Gaetano Pini 9, Centro Specialistico Ortopedico Traumatologico, Gaetano Pini-CTO, Milano, Italy
2. **Dr. Gianluca Sambataro**, Medico Immunologia e reumatologia presso, Artoreuma S.R.L., Cors S. Vito 53, 95030 Mascalucia, CT, Italy
3. **Dr. Atzeni Fabiola**,  Full Professor, Rheumatology Unit,  University of Messina, Messina, Italy
4. **Dr. Marcello Govoni**, Professor, Department of Medical Sciences, Complex Operative Unit and Rheumatology Unit of S.Anna University Hospital, University of Ferrara, Via A. Moro 8, 44124- Cona (FE), Italy
5. **Dr Simone Parisi**, Epidemiology Unit, Italian Society for Rheumatology, Milan, Italy; Rheumatology Unit, Azienda Ospedaliera Città della Salute e della Scienza di Torino, Torino, Italy
6. **Dr Elena Bartoloni Bocci**, Associate Professor, Department of Medicine and Surgery, MED/16- Rheumatology, Università degli studi di Perugia, P.zza Università - 06123 – Perugia, Italy
7. **Dr. Gian Domenico Sebastiani**, U.O.C. Reumatologia, Ospedale San Camillo-Forlanini, Roma, Italy
8. **Dr Enrico Fusaro**, Rheumatology Unit, Azienda Ospedaliero-Universitaria Città della Salute e della Scienza di Torino, Torino, Italy
9. **Dr Marco Sebastiani**, Rheumatology Unit, University of Modena and Reggio Emilia, Azienda Ospedaliero-Universitaria Policlinico di Modena, Via del Pozzo, 41125, Modena, Italy
10. **Dr Luca Quartuccio**, Clinic of Rheumatology, Department of Medicine (DAME), ASUFC, University of Udine, Udine, Italy.
11. **Dr Franco Franceschini**, Rheumatology and Clinical Immunology Unit, Department of Clinical and Experimental Sciences, ASST Spedali Civili and University of Brescia, Italy
12. **Dr Pier Paolo Sainaghi**, Department of Translational Medicine, Università del Piemonte Orientale UPO, Novara, Italy; Division of Internal Medicine, Immunorheumatology Unit, CAAD (Center for Translational Research on Autoimmune and Allergic Disease) Maggiore della Carità Hospital, Novara, Italy; IRCAD, Interdisciplinary Research Center of Autoimmune Diseases, Novara.
13. **Dr Giovanni Orsolini**, Department of Medicine, Rheumatology Unit, University of Verona, Verona, Italy
14. **Dr Rossella De Angelis**, Rossella De Angelis, Rheumatology Unit, Department of Clinical and Molecular Sciences, Polytechnic University of Marche
15. **Dr Maria Giovanna Danielli**, Clinica Medica, Dipartimento di Scienze Cliniche e Molecolari, Università Politecnica delle Marche e Azienda Ospedali Riuniti, Ancona, Italy.
16. **Dr Vincenzo Venerito**, Department of Emergency and Organ Transplantations-Rheumatology Unit, University of Bari "Aldo Moro", Bari, Italy

**Spain**

1. **Dr Jesús Loarce-Martos**, Rheumatology Department, Hospital Universitario Ramón y Cajal, Carretera de Colmenar Viejo, 9, 1 km, 28043, Madrid, Spain.
2. **Dr Sergio Prieto-González**, Department of Internal Medicine, Hospital Clinic of Barcelona, University of Barcelona, Barcelona, Spain.
3. **Dr Albert Gil-Vila,** Systemic Autoimmune Diseases Unit, Vall d'Hebron General Hospital, Medicine Dept, Universitat Autónoma de Barcelona, Barcelona, Spain
4. **Dr Raquel Aranega Gonzalez,** Systemic Autoimmune Diseases Unit, Vall d'Hebron General Hospital, Medicine Dept, Universitat Autónoma de Barcelona, Barcelona, Spain

**Japan**

1. **Dr Ran Nakashima**, Department of Rheumatology and Clinical Immunology, Graduate School of Medicine, Kyoto University, 54 Shogoin-Kawahara-cho, Sakyo-ku, Kyoto 606-8507, Japan.
2. **Dr Shinji Sato**, Division of Rheumatology, Department of Internal Medicine, Tokai University School of Medicine, 143 Shimokasuya, Isehara, 259-1193, Japan.
3. **Dr Naoki Kimura**, Department of Lifetime Clinical Immunology, Graduate School of Medical and Dental Sciences, Tokyo Medical and Dental University (TMDU), Tokyo, Japan
4. **Dr Yuko Kaneko**, Division of Rheumatology, Department of Internal Medicine, Keio University School of Medicine, Tokyo, Japan.

**Germany**

1. **Dr Stylianos Tomaras**, Department of Rheumatology, Helios Clinic Vogelsang-Gommern, 39245 Gommern, Germany

**Russian Federation**

1. **Dr Margarita Aleksandrovna Gromova**, Pirogov Russian National Research Medical University (RNRMU), Moscow, Russian Federation.

**Israel**

1. **Mr Or Aharonov**, Department of Gerontology, Faculty of Social Welfare and Health Science, University of Haifa, Haifa, Israel.

**Morocco**

1. **Dr Ihsane Hmamouchi**, Faculty of Medicine, Laboratory of Clinical Research and Epidemiology, Mohammed V University, Rabat, Morocco.

Other Investigators

**France**

1. **Dr. Margherita Giannini**, Explorations fonctionnelles musculaires, service de physiologie, Hôpitaux universitaires de Strasbourg; EA3072, fédération de médecine translationnelle.
2. **Dr François Maurier-** Service de Médecine Interne, Hôspital Robert Schuman, Rue de Champ Montoy, 57070 Vantoux, France.
3. **Dr Julien Campagne-** Service de Médecine Interne, Hôspital Robert Schuman, Rue de Champ Montoy, 57070 Vantoux, France.
4. **Dr Alain Meyer-** 1. Centre National de Référence des Maladies Systémiques et Auto-immunes Rares Grand-Est Sud-Ouest (RESO), Service de humatologie, Service de physiologie, Unité d’explorations fonctionnelles musculaires, Hôpitaux Universitaires de Strasbourg, Strasbourg, France; 2. EA3072, Fédération de Médecine Translationelle, Université de Strasbourg, Strasbourg, France.

**Hungary**

1. **Dr Melinda Nagy-Vincze**- 1. Division of Clinical Immunology, Faculty of Medicine, University of Debrecen, Móricz Zsigmond út 22, Debrecen, H-4032, Hungary. 2. Gyula Petrányi Doctoral School of Clinical Immunology and Allergology, University of Debrecen, Debrecen, Hungary.

**Australia**

1. **Dr Daman Langguth**- Department of Immunology, Sullivan Nicolaides Pathology, Brisbane, Queensland, Australia.
2. **Dr Vidya Limaye**- Consultant Rheumatologist, Royal Adelaide Hospital, Associate Professor of Rheumatology, Discipline of Medicine, University of Adelaide, Australia.
3. **Dr Merrilee Needham**- 1. Neurology Department, Fiona Stanley Hospital, Murdoch, Australia; 2. Institute for Immunology and Infectious Diseases, Murdoch University, Murdoch, Australia; 3. Perron Institute for Neurological and Translational Science, Nedlands, Australia; 4. University of Notre Dame, Fremantle, Australia.
4. **Dr Nilesh Srivastav**- Alfred Health, The Alfred, Caulfield Hospital, Sandringham Hospital, Melbourne, Victoria, Australia.

**Canada**

1. **Dr Marie Hudson-** Department of Medicine, McGill University, Montreal, Quebec; Division of Rheumatology, Jewish General Hospital, Montreal, Quebec, and Lady Davis Institute, Jewish General Hospital, Montreal, Quebec, Canada.
2. **Dr Océane Landon-Cardinal-** Department of Medicine, University of Montreal, Montreal, Canada. Department of Medicine, CHUM Research Centre, Montreal, Canada.

**Malaysia**

- - - 1. **Dr. Syahrul Sazliyana Shaharir**- Rheumatology Unit, Department of Internal Medicine, Universiti Kebangsaan Malaysia Medical Centre (UKMMC), Jalan Yaacob Latiff, Kuala Lumpur, Malaysia

**Columbia**

- - - 1. **Dr Wilmer Gerardo Rojas Zuleta**- Department of Rheumatology, Universidad de Antioquia, Cl. 67 #53 - 108, Medellín, Colombia.

**Portugal**

**Dr José António Pereira Silva**- Rheumatology Department, Centro Hospitalar e Universitário de Coimbra EPE, and Coimbra Institute of Clinical and Biomedical Research (iCBR), Faculty of Medicine, University of Coimbra, Portugal.

**Dr João Eurico Fonseca**- Hospital de Santa Maria, Centro Hospitalar Lisboa Norte Centro Académico de Medicina de Lisboa, Lisboa, Portugal; Instituto de Medicina Molecular, Faculdade de Medicina, Universidade de Lisboa, Lisboa, Portugal.

**Ukraine**

**Dr Olena Zimba-** Department of Internal Medicine #2, Danylo Halytsky Lviv National Medical University, Lviv, Ukraine.
